# Systems Biology in the Context of Big Data and Networks

**DOI:** 10.1155/2014/428570

**Published:** 2014-05-27

**Authors:** Md. Altaf-Ul-Amin, Farit Mochamad Afendi, Samuel Kuria Kiboi, Shigehiko Kanaya

**Affiliations:** ^1^Nara Institute of Science and Technology, Japan; ^2^Bogor Agricultural University, Indonesia; ^3^University of Nairobi, Kenya

## Abstract

Science is going through two rapidly changing phenomena: one is the increasing capabilities of the computers and software tools from terabytes to petabytes and beyond, and the other is the advancement in high-throughput molecular biology producing piles of data related to genomes, transcriptomes, proteomes, metabolomes, interactomes, and so on. Biology has become a data intensive science and as a consequence biology and computer science have become complementary to each other bridged by other branches of science such as statistics, mathematics, physics, and chemistry. The combination of versatile knowledge has caused the advent of big-data biology, network biology, and other new branches of biology. Network biology for instance facilitates the system-level understanding of the cell or cellular components and subprocesses. It is often also referred to as systems biology. The purpose of this field is to understand organisms or cells as a whole at various levels of functions and mechanisms. Systems biology is now facing the challenges of analyzing big molecular biological data and huge biological networks. This review gives an overview of the progress in big-data biology, and data handling and also introduces some applications of networks and multivariate analysis in systems biology.

## 1. Introduction

Biology has recently become a “big-data science” mainly supported by the advances in high-throughput experimental technologies. Data-intensive science consists of three basic activities: capture, curation, and analysis [1]. All three of these phases of handling big data raise many new research challenges to pursue in systems biology. The big data challenges are not only their size but also their increasing complexity. The emergence of big data biological sciences, such as systems biology, and their growing impact on health, nutrition, ecosystems, and other societal issues have only recently become the focus of scholars in social studies, science, and information studies [2]. Jim Gray proposed the fourth data paradigm and farming of the “data deluge;” that is, the capacity to measure, store, analyze, and visualize data is the new reality to which science must adapt. The heart of the fourth paradigm is data and it sits alongside empiricism (1st paradigm), theory (2nd paradigm), and simulation (3rd paradigm), which together form the continuum we think of as the modern scientific method [1]. Systems biology is one of the several other subjects including astronomy, ecology, and meteorology where challenges of the fourth data paradigm have become relevant. The basic purpose of systems biology is the system-level understanding of a cell or an organism, which can be summarized in the context of molecular networks as (1) an understanding of the structure of all the components of a cell/organism up to molecular level, (2) the ability to predict the future state of the cell/organism under a normal environment, (3) the ability to predict the output responses for a given input stimulus, and (4) the ability to estimate the changes in system behavior upon perturbation of the components or the environment. In a cell or organism the primary-level components, for example, the molecules, are of numerous types and numbers and hence system-level understanding of a cell/organism is still a very difficult task. However along the way to achieve the theoretical goal of systems biology, that is, to understand life scientifically, many other practical applications will be invented. Practical applications will include development of new generation medical tests, drugs, foods, fuel, materials, sensors, and other applications. Systems biology now faces the challenges of analyzing large amounts of molecular biological data and huge biological networks.

## 2. Big Picture of Hierarchy and Networks in Systems Biology

**Figure 1:**
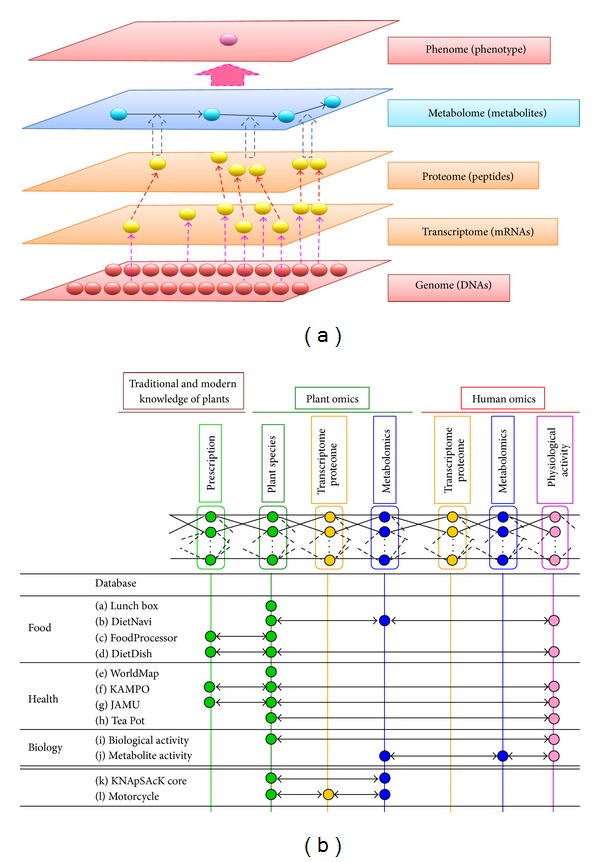
(a) Hierarchy of the omics molecules and (b) integrated platform of knowledge in KNApSAcK database for plants and plant and human omics.

The hierarchy shown in Figure 1(a) summarizes the major types of molecules being studied in systems biology, which aims to determine the functions of the molecules of each layer and how these molecules interact with each other within individual layers and between layers to perform biological tasks.  Figure 1(b) shows the overview of the accumulated data in the KNApSAcK database which has been developed to facilitate the knowledge discovery regarding plants and plant-human omics [3]. The upper part of Figure 1(a) can be regarded as an example of a big picture of networks in systems biology. This figure implies the existence and abstraction of networks in individual species and across species. Numerous studies constructed suitable networks for understanding systems or subsystems within species. Networks representing systems or subsystems can also be compared or linked between species (Figure 1(b)). This world is cohabitated by humans and many other species and the understanding of the interactions at the molecular level among all the species is important for healthy and sustainable living for humans and other organisms.

## 3. Data Types in Systems Biology

Many experiments are conducted in systems biology like many other branches of science; these experiments produce various types of data. Currently in systems biology some of the popularly-used data types are as follows.

### 3.1. Sequences

The DNA is a molecule of double helix structure that consisted of two complementary strands of sequences of four nucleotide bases—adenine, thymine, guanine, and cytosine, represented as A, T, G, and C, respectively [4]. DNA contains all the necessary information preserved in the order of the nucleotide sequences. Hence, it is important to determine the sequences accurately. A gene is usually a continuous part of one of the DNA strands and contains codes for one or a few different proteins. The proteins are essential molecules that consisted of amino acid sequences. From the starting site of a gene, every three nucleotides are called a codon and a codon corresponds to an amino acid. It is in this way that a gene preserves the code of a protein. For example ATGAAGCTACTGTCTTCTATCGAACAAGCATGCGAT is the sequence of the first 30 nucleotides of GAL4 gene of yeast and KLLSSIEQAC is the sequence of the first 10 amino acids of the corresponding protein. There is variation in codon usage by different organisms and links can be established between codon usage and the biological characteristics of an organism [5, 6]. Development of DNA sequencing techniques started in the 1970s and since then various methods have been developed [7–9]. The sequence of individual genes, group of genes, parts of chromosomes, full chromosomes, or entire genomes are determined for different purposes. Recent developments in next generation sequencing techniques have greatly reduced the time and cost of sequencing [10–12].

### 3.2. Molecular Structure

Determination and prediction of the three-dimensional structures of omics molecules are also very important. DNA is packed into protein-DNA structures referred to as chromatin mainly to fit the long DNA chain inside the cell. The primary protein components of chromatin are histones. The DNA packaging protects DNA from damage and plays important roles in gene regulation by allowing or blocking the binding of transcription factors and other molecules to DNA. Usually, proteins also work by forming complexes with other proteins. In general it can be stated that DNA, RNA, and protein molecules usually bind with one another dynamically to perform different cellular functions. Therefore, not only the sequences but also the three-dimensional structures of the omics molecules are important for predicting the possibility of binding between molecules and thus predict the functions of uncharacterized molecules. X-ray crystallography, nuclear magnetic resonance (NMR), and electron microscopy are the experimental procedures used for determining the 3D structures of proteins. A number of methods for the computational prediction of protein structure from its sequence have been developed [13, 14]. Also, there are computational methods for the prediction of RNA structures [15–17]. There are numerous software tools for predicting and visualizing 3D structures of proteins and RNAs. A comprehensive list of these tools can be found in scientific literature. Molecular structure data are therefore three-dimensional geometrical figures of versatile shapes or related information that can be easily converted to three-dimensional structures usually with the aid of computer software.

### 3.3. Gene Expression

Gene expression is the process of extracting information of a gene and is the initial step of producing gene products such as mRNAs which are usually translated to proteins and functional RNAs such as rRNA or tRNA. Gene expression is known to take place in all life forms, that is, eukaryotes (unicellular and multicellular), prokaryotes (bacteria and Archaea), and viruses—to generate the macromolecular machinery and building blocks for life. Though most cells in an organism contain the same genes, not all of the genes are used in each cell. Some genes are turned on, or “expressed,” when needed in particular types of cells. Microarray technology [18, 19] allows us to look at many genes at once and determine which are expressed in a particular cell type and to what extent. Next generation sequencers are also currently used to determine the gene expression [20]. To say that “a gene is highly expressed” means many copies of mRNA corresponding to that gene are produced in the cell. The extent of expression of genes is usually measured for comparison by using samples collected under different experimental conditions, for example, sick and healthy tissues, normal cells or cells put under certain stress or starving. Gene expression data is usually represented as a matrix where the rows represent genes and the columns represent experimental conditions; that is, gene expression data are multivariate data.

### 3.4. Binding Sites and Domains

Many important cell processes such as RNA transcription, DNA packing, DNA replication, DNA recombination, and DNA repair are initiated and regulated by binding of proteins to selected DNA sequences. A position weight matrix (PWM) is a commonly-used representation of motifs (patterns) in biological sequences [21]. A PWM, also called position-specific weight matrix (PSWM) or position-specific scoring matrix (PSSM), is a matrix of score values that gives a weighted match to any given substring of fixed length. A DNA-binding domain (DBD) of a protein is an independently folded domain that contains at least one motif that identifies and binds double- or single-stranded DNA. A DBD can recognize a specific DNA sequence usually known as a recognition sequence or have a general affinity to DNA [22]. The domains of proteins and the binding sites at DNA are therefore part of the sequences of the corresponding proteins and the DNA, respectively.

### 3.5. Protein-Protein Interaction (PPI)

In cells, thousands of different types of proteins act as enzymes-catalysts to chemical reactions of the metabolism, components of cellular machinery (e.g., ribosomes), regulators of gene expression, and so on. Some proteins play specific roles in special cellular compartments, whereas others move from one compartment to another carrying mass or information. A protein may work as an individual entity, but usually two or more proteins bind together and form a complex to carry out their biological functions. The RNA polymerase, a large molecular machine that copies information from DNA to produce mRNA, is indeed a big protein complex that consisted of many proteins. Proteins are generally bound together in a complex not by chemical bonds but by other forces. Usually PPI data are represented as binary relation between two proteins whether they are part of two-protein complex or multi-protein complex. All or a number of the PPIs of an organism can be represented as a network where a protein represents a node and an interaction represents an edge. Experiments that are used to determine PPIs are yeast two hybrid system (Y2H) [23, 24], affinity purification coupled to MS (AP-MS) [25], and so forth.

### 3.6. Mass Spectrometry

Mass spectrometry (MS) is an analytical technique that produces spectra (singular spectrum) of the masses of the molecules comprising a sample. The spectra are used to determine the elemental composition of a sample, the masses of particles and of molecules, and to elucidate the chemical structures of molecules, such as peptides, metabolites, and other chemical compounds. Mass spectrometry works by ionizing chemical compounds to generate charged molecules or molecule fragments and measuring their mass-to-charge ratios [26]. Mass spectrometry data can be represented as 2- (molecular weight versus magnitude) or 3- (molecular weight versus magnitude versus time) dimensional arrays; that is, they can be treated as multivariate data.

### 3.7. Metabolic Pathways

Living cells generate energy and produce building material for cell components and replenishing enzymes by the process of metabolism. All organisms live and grow by receiving food and nutrients from the environment. The foods are processed through thousands of reactions. In cells chemical reactions take place around-the-clock, constantly breaking and making chemical molecules and transferring ions and electrons. These reactions are called metabolic pathways. All or a group of known metabolic reactions of an organism can be represented as a network where metabolites are considered as nodes and a reaction between them is represented as edges. The edges in metabolic pathways correspond to one or more enzymes. Metabolic reactions follow the laws of physics and chemistry and thus modeling of metabolic reactions requires considering many physicochemical constraints [27]. In summary, it can be said that in terms of structure, extensively-used data in systems biology consist of four types: sequence data, 3D-structure data, multivariate data, and network data. However, the present challenge is that the amount of data is expanding rapidly requiring new tools and algorithms for handling big data. One type of data can be converted to another type for convenience of analysis. In the following section we discuss how networks can be generated from multivariate data and sequence data.

## 4. Network Generation from Different Data Types

In multivariate data, entities are represented by multiple variables and each entity can be regarded as a point in a multidimensional space or as a profile wave sketched according to the data values. Therefore, to convert multivariate data to a network, it is necessary to use a metric or some kind of measure that can assess distance or similarity between two multivariate entities. Widely-used distance or similarity measures are Euclidean distance, Manhattan distance, Mahalanobis distance, Correlation, and so forth [28–30]. The value of correlation ranges from −1 to +1 and the higher the value between two multivariate entities the more similar the entities. The opposite of distance can be used as a measure of similarity. Usually similarity between each pair of entities is calculated and then a threshold similarity is decided based on statistical analysis or some other important criteria, for example, to ensure scale-free degree distribution of the generated network or something like that. After selecting the threshold, all entities of the multivariate data are considered as the nodes of a network and an edge is inserted between the pair of entities for which the similarity is more than the threshold. A weighted network can be constructed by considering the similarity values as the weights of the edges. Sometimes one type of network is converted to another type for the convenience of applying algorithms or for some other purposes. In [31] the metabolic pathways are converted to a simple network of enzymes/genes. After that, graph spectral clustering was applied to the converted networks corresponding to* M. tuberculosis*,* M. leprae,* and* E. coli*. It was observed that reactions belonging to fatty acid biosynthesis and the FAS-II cycle of the mycolic acid pathway in* M. tuberculosis* form distinct, tightly connected subclusters. Also, based on degree centrality and eigenvector centrality the important genes in the networks were determined and their functions were analyzed. In [32] a PPI network was converted to the corresponding line graph for the convenience of applying a clustering algorithm. The conversion to line graph helped to place the related proteins to densely connected regions or clusters and thus paved the way to obtain useful results by the application of a graph clustering algorithm.

## 5. Big Biological Databases

Curation and analysis become important after capturing data from various experiments. Curation includes storage, retrieval, spreading around the world, filtering and integrating the data. The engineering techniques for these jobs are already known, but when that data is in the petabyte scale, it becomes complicated. Algorithms and software tools developed for the analysis of biological data also face the problems of scalability when data becomes very big. However, many big databases have been created around the world for curation and analysis of biological data and their data volume and performance are gradually improving. DNA Data Bank of Japan [33] and GenBank [34] are big databases of primary nucleotide sequences of many organisms which are related to the bottom level (Genome) of the hierarchy shown in Figure 1(a). PGDBj is a portal website for the integration of plant genome-related databases [35]. Gene expression omnibus (GEO) from NCBI is a data repository of array- or sequence-based gene expression profiles. ATTED-II is a database of coexpressed genes [36]. Information about noncoding RNA (ncRNA) families and other structured RNA elements can be found in Rfam database [37]. For the sequences and annotations of microRNAs, a useful database is miRBase [38]. GEO, ATTED-II, Rfam, and miRBase are related to the transcriptome level of Figure 1(a). UniProt is a comprehensive and freely accessible database of protein sequences and functional information of proteins [39]. The PROSITE database [40] consists of entries describing the protein families, domains, and functional sites as well as amino acid patterns and profiles of them. BIND [41] and BioGRID [42] are databases of protein-protein interactions. UniProt, PROSITE, BIND, and BioGRID are related to the proteome level of Figure 1(a). A central archive of macromolecular structural data is wwPDB [43]. The data accumulated in wwPDB is freely and publicly available to the global community. There are four member sites of wwPDB as follows: RCSB PDB (USA), PDBe (Europe), PDBj (Japan), and BMRB (USA). NetPath [44] is a manually curated database of signal transduction pathways in human. For metabolic pathways KEGG is a rich and well known database. KNApSAcK is a metabolomics database which was initially developed as a species metabolite relational database [45] and afterwards extended to KNApSAcK family databases containing information about herbal medicines [46, 47] and metabolite activities [3]. KEGG and KNApSAcK are mainly associated with metabolome level of Figure 1(a). A comprehensive list of the omics databases can be found by searching the internet with the term “list of biological databases.”

## 6. Multivariate Analysis in Systems Biology

After capture and curation of data, the next step is analysis. Algorithms for analyzing multivariate data developed for other applications are currently used extensively in systems biology. The well-known methods for handling multivariate data are related to dimension reduction, clustering, classification, and regression. Often, dimension reduction is done before applying other methods. Principal component analysis (PCA) is the popular algorithm for dimension reduction [48]. PCA is a mathematical process that converts the values of a set of possibly-correlated variables into a set of values of uncorrelated variables which are called principal components. This transformation assigns the largest possible variance to the first principal component and usually the sum of variance of first few components approaches the total variance of all the variables in the original data. Therefore, variable reduction is performed by replacing all the original variables by the first few components obtained from PCA analysis.

Regression analysis is a process for estimating the relationships between dependent variables (response variable) and independent (predictor) variables. Most regression analysis techniques estimate coefficients to establish a linear relation between dependent and independent variables. Least squares regression [49] and partial least squares (PLS) regression [50] are popular regression techniques. In multivariate data analysis, classification is the problem of identifying the category of a new observation from among a set of categories. Support vector machine (SVM) is a popular algorithm for classifying multivariate entities into two categories [51]. A multivariate entity can be regarded as a point in a multidimensional space. Usually an optimum hyperplane is determined based on training data so that multivariate entities of one category fall on one side of the hyperplane, while the entities of the other category fall on the other side. The concept of SVM can be extended to classify multivariate entities into multiple categories. Another type of classifier is the neural network [52], which is a naïve way of electronically simulating the function of the human brain. It is difficult to make a single formal definition of all the methods considered neural networks in the scientific literature. Usually, a neural network consists of a layer of input nodes and a layer of output nodes and several hidden layers of nodes. A neural network can be trained to use it as a classifier of multivariate entities. A multivariate data vector can be applied to the input nodes and after mathematically processing values applied at the input nodes by functions associated to the hidden nodes some values are propagated to the output nodes, which are utilized to determine the class of the input multivariate entity. The functions associated to the hidden nodes are determined or optimized based on the training data. The naïve Bayes classifier [53] is another popular supervised classification technique applicable to multivariate data. This classification algorithm is named after Thomas Bayes (1702–1761), who proposed the Bayes theorem. However it is called naïve Bayes because it naively assumes that the features or variables that describe a multivariate entity are mutually independent. Naïve Bayes classifier usually computes the probability that a multivariate entity belongs to a certain class given its features. Usually a set of training data or well-defined probability density functions are used to estimate different probabilities required to classify a multivariate entity. Random forest [54] is another classification method. The random forest is an ensemble classifier which constructs multiple decision trees. Each tree is constructed using a subset of training data and a subset of variables. Class assignment is made by the number of votes from all of the trees. Random forests can also be used to rank the importance of the variables in a regression or classification problem. Some other classification algorithms are partial least squares discriminant analysis (PLS-DA) [55] and soft independent modeling of class analogy (SIMCA) [56].

Another multivariate technique common in systems biology is clustering. This is the task of dividing a set of entities or objects into several groups or clusters in such a way that the objects in the same cluster are more similar in some sense to each other than to those in other clusters. Clustering and classification are related concepts, but in the case of classification, the categories are known beforehand, whereas in case of clustering, usually the categories are understood after applying a clustering algorithm. Hierarchical clustering [57, 58] is the widely used algorithm for clustering of multivariate data. Hierarchical clustering is subdivided into 2 types: agglomerative methods and divisive methods. Agglomerative methods proceed by a series of fusions of the objects into groups eventually encompassing all objects in a single group. On the other hand, the divisive method separates the objects successively into finer groupings, eventually keeping each object in a single group. Hierarchical clustering is a technique that organizes elements into a tree.* K*-mean clustering [59] and self-organizing mapping (SOM) [60, 61] are also important clustering algorithms applicable to multivariate data.* K*-mean is one of the simplest unsupervised clustering methods. One disadvantage of* K*-mean clustering is that it is necessary to guess and set the number of clusters in the targeted dataset before applying the algorithm. In case of SOM, multidimensional data/input vectors are mapped onto a two-dimensional array of nodes. Data points assigned to a node or nearby nodes are considered as a cluster.

Data assimilation can be referred to as state estimation which is the process of combining a model with observational data to estimate the state of a system. By data assimilation, a quantity of interest is estimated by combining observational data with the underlying dynamical principles governing the system under investigation. There are applications of data assimilations in systems biology. The data assimilation technique was applied to elucidate the dynamics of time-lagged gene-to-metabolite networks of* Escherichia coli* [62]. State transitions in the transcriptome of* Bacillus subtilis* and in both transcriptome and metabolome of* Arabidopsis thaliana* were predicted using a data assimilation technique called linear dynamical system model [63].

Numerous researches have been conducted in systems biology based on multivariate data analysis. We briefly discuss a few examples below.

### 6.1. Application of BL-SOM

Batch learning self-organizing map (BL-SOM) is a novel neural-network algorithm that has been applied to efficiently and comprehensively analyze codon usage in approximately 60,000 genes from 29 bacterial species simultaneously [61]. In the original SOM method [60], the initial weight vectors are set by random values, but in BL-SOM the vectors are initialized by PCA, which is a statistical method that performs linear mapping to extract optimal features from an input distribution in the mean squared error sense. This technique allows the resulting SOM to be independent of input vectors. BL-SOM makes it possible to cluster and visualize the genes of individual species separately at a much higher resolution than can be obtained with PCA because PCA works based on linear mechanism while SOM can be trained to adapt non-linear mechanisms. The organization of the SOM can be explained by the genome G + C and tRNA compositions of the individual species. This work further used SOM to examine codon usage heterogeneity in the* E. coli* O157 genome, which contains “O157-unique segments” (O-islands), and showed that SOM is a powerful tool for characterization of horizontally transferred genes. Another example of the application of BL-SOM is the investigation of the enzyme sequence diversity related to secondary metabolism [64]. Initially, a map was constructed by using a big data matrix that consisted of the frequencies of all possible dipeptides in the protein sequence segments of plants and bacteria. The enzyme sequence diversity of the secondary metabolic pathways was examined by identifying clusters of segments associated with certain enzyme groups in the resulting map. The extent of diversity of fifteen secondary metabolic enzyme groups was discussed. On the resulting map, six clusters were rich with fragments of monoterpene, sesquiterpene, diterpene, and triterpene synthases. Nine clusters are corresponding to eight types of phenylpropanoids which are flavonoid and isoflavonoid synthases. Five clusters were associated to acetyl-, O-methyl-, and N-methyl transferases. As a whole these results show sequence similarities between specific types of enzymes related to secondary metabolic pathways.

### 6.2. Application of PLS-DA Model

PLS-DA is an example of a multivariate model that has been applied in systems biology a case study being our previous work on Indonesian herbal medicines, popularly known as Jamu. These medicines are prepared from a mixture of several plants. The plants are chosen so that the Jamu has the desired efficacy. Thus, the composition of the plants used in a Jamu formula determines its efficacy. A model using partial least square discriminant analysis (PLS-DA) has been developed to predict the efficacy of Jamu based on the information of plants used in Jamu formula [55]. In this analysis, among 3,138 Jamu medicines, the efficacies of 2,248 Jamu medicines (71.6) were correctly predicted. Hence, the efficacy in most Jamu medicines can be predicted on the basis of the ingredient medicinal plants. In addition, the regression coefficients of the PLS-DA model, which relates plant usage in Jamu as predictors and Jamu efficacy as response, can be helpful in determining which plants in the ingredients of Jamu are used as main ingredients, which contribute primarily to the medicines' efficacies, and which plants are used as supporting ingredients. Plants that act as main ingredients will have a significant effect on the developed model. Due to the absence of parametric testing for the PLS-DA coefficients, the evaluation for significance is performed using permutation testing, in which the distribution of coefficients under the null hypothesis is generated via resampling of the existing data. The resampling is performed by permuting the order of the responses (in this case, Jamu efficacies) while maintaining the order of the predictors (in this case, plant utilization as Jamu ingredients) so that the existing relationship between the predictors and the response is destroyed and a new data set is generated under the null hypothesis; that is, plant utilization in Jamu does not affect Jamu efficacy. If such resampling is performed many times and the PLS-DA model is applied on the new data generated from the resampling, the accumulation of the PLS-DA coefficients obtained from this process generates a distribution, against which a *P* value can be calculated and subsequently evaluated for significance. From the testing, it was observed that 234 plants (50.3 among all 465 plants) showed no significant status for all 9 efficacies; whereas the other 231 plants have a significant status of which 189 plants (40.6) are significant only for 1 efficacy, 38 plants (8.2) are significant for 2 efficacies, and the other 4 plants (0.9) are significant for 3 efficacies.

## 7. Network Analysis in Systems Biology

For system-level understanding, initially the elements of a system are connected based on their mutual relation and a network is formed. Global network properties such as average path length, clustering coefficient, and degree distribution [65] are determined to assess the overall characteristics of the network such as how they are formed, what model they fit, how robust they are, and how tightly the elements are connected. There are numerous algorithms for finding clusters in a network. As a flexible notion the densely connected regions of a network are called clusters. Also, there are precise definitions of network clusters such as k-core, k-plex, and n-clan [66–68]. In recent years network theory has been substantially applied in systems biology. Construction and analysis of biological networks have become highly popular among omics researchers. In the following section we discuss some of the applications of networks and network algorithms in systems biology.

### 7.1. Function Prediction

Functions of many omics molecules or entities, for example, genes, mRNAs, proteins, and also metabolites, are still unknown. A system-level approach of predicting functions of an unknown entity is performed by constructing a network of that entity and other known and unknown entities. Usually, after constructing a network, some suitable clustering method is applied. There are versatile graph clustering methods such as based on density and periphery [69], random walk [70], betweenness centrality [71], and so on. Usually the entities belonging to the same cluster are considered to have similar function based on the hypothesis “guilt by association” and therefore if the majority of the members of a cluster have some known function, then the unknown members are also assumed to have that function.

### 7.2. Protein Complex Detection

Protein molecules may act individually, but in most cases to perform a biological task they form complexes by binding with one or more other protein molecules. High throughput experiments such as yeast two hybrid system (Y2H) [23, 24] and affinity purification coupled to MS (AP-MS) [25] are used to determine the global set of interacting protein pairs. Such protein pairs can be represented as a network which is known as a PPI network. Usually it is assumed that a set of proteins in a densely connected region in a PPI network correspond to a protein complex. A good number of researches have been conducted to computationally detect protein complexes by applying clustering algorithms to PPI networks [72–76]. In those studies it was shown that real protein complexes of yeast substantially matched with computationally detected protein complexes.

### 7.3. Prediction of Interaction

The presence of statistically significant complementary domain pairs in interacting protein pairs determined in the context of a PPI network indicates that certain domains facilitate protein binding [77, 78]. Thus the presence of complementary domains in two new proteins implies the possibility that they might interact inside the cell. Thus, PPI networks of one or more species can be used to first determine complementary domain pairs and then to predict interactions between new protein pairs corresponding to a species.

### 7.4. Analyzing Evolution

PathBLAST [79] is a network alignment and search tool for comparing protein interaction networks across species to identify protein pathways and complexes that have been conserved by evolution. The basic method searches for high-scoring alignments between pairs of protein interaction paths, for which proteins of the first path are paired with putative orthologs occurring in the same order in the second path.

### 7.5. Information Integration

Networks can be constructed by combining different types of information, thus being helpful for integrated analysis of different omics molecules based on their relations. An integrative network of* C. elegans* embryogenesis genes based on three types of data (protein-protein interaction, expression profiling similarity, and phenotyping profiling similarity) was studied in [80]. This study showed that gene pairs connected by interactions supported by multiple data are more likely to belong to the same GO category. For example in [81] gene expression profiles and mass spectrometry profiles are merged by using appropriate normalization of the data and a combined network of genes and metabolites has been constructed which helped find related genes and metabolites. A very large network of more than 60,000 interactions was reported [82] by integrating transcription factor binding, PPI, and protein phosphorylation data of yeast. This network was found to contain 7 types of 3-molecule motifs involving kinases out of which 5 types were overrepresented.

### 7.6. Determination of Important Entities

It is easy to realize that in the context of a network all nodes are not equally important. For example, a node with very high degree is obviously more important compared to a node having degree 1 or 2. There is an important relation between vertex degree and functional importance of the vertices in biological networks [83]. It has been reported that in PPI networks the removal of highly connected proteins is more likely to have more lethal effect [84]. The importance of a node in a network is precisely and mathematically determined by the centrality measures, for example, degree centrality, closeness centrality, betweenness centrality, eigenvector centrality, and so forth. In [85] a list and definitions of 17 types of different centrality measures are presented.

### 7.7. Disease Diagnosis

Biological networks can be utilized to identify biomarkers for disease diagnosis. Even a subnetwork also might be a biomarker. Protein network and mRNA profiles can be integrated to identify subnetwork biomarkers, that is, highly connected genes of a subnetwork whose sum of expression can be a marker of a disease state. There are several network-based approaches for identifying disease genes and protein interaction subnetworks which are disease signatures [86–88]. The application of a network analysis to metabolic PET (positron emission tomography) data obtained from patients with Parkinson's disease resulted in the identification and validation of two distinct spatial covariance patterns associated with the motor and cognitive manifestations of the disease [89].

### 7.8. Drug Development

Complicated diseases such as cancer, Alzheimer, mental disorder, and heart diseases are very complex and caused by multiple molecular abnormalities. The drug discovery process of these diseases needs to target not a single molecule but entire molecular pathways of various cellular omics networks. Recently biological networks, for example, PPI networks and gene expression networks, are extensively used to find drug targets [90–92]. In [93], a method for drug target identification was proposed by combining information about drug therapeutic similarity, chemical similarity, and protein-protein interaction network using linear regression.

### 7.9. Prediction of Drug-Drug Interactions

Understanding drug-drug interaction is important for drug development and drug administration. A drug interaction is a situation in which a substance (usually a drug) affects the activity of another drug when both are administered together. Drug-drug interaction is a significant cause of adverse drug reaction, especially in population on multiple medications. Drug-drug interaction can be categorized into three types: pharmaceutical, pharmacokinetic (PK), and pharmacodynamic (PD). A prediction method of pharmacodynamic drug-drug interaction through protein-protein interaction networks is proposed in [94]. This work introduced a metric called “S-score” that measures the strength of network connection between drug targets. Thus drug-drug interaction was determined by assessing the interaction between the drug targets in the context of the whole PPI network.

### 7.10. Comparison of Biological Mechanisms

Different types of biological networks, for example, PPI networks, gene regulatory networks, and metabolic pathways, and so forth, are system-level representations of biological mechanisms. Interesting results were obtained by comparing biological networks with random networks of the same size [69, 95] or biological networks derived under different contexts [96]. Usually such comparisons are performed in the context of global network properties like degree distribution, average path length, and clustering coefficient, and so forth. Though global level degree distribution of PPI networks of many species follows power law, subtle differences between PPI networks of different species can be found by using other concepts. Not only PPI network but also other types of biological networks of different species can be compared to decipher the differences in mechanisms to explain phenotypes and other useful matters. A distance measure called relative graphlet frequency distance is presented in [97] which is based on the frequency of undirected induced subgraphs of size three to five. This measure was used to compare PPI networks of* E. coli* and yeast with different artificial networks [98]. Another concept of comparing two networks especially regulatory networks is on the basis of network motifs which are reoccurring patterns in complex networks and thus in some sense similar to the motifs in gene or protein sequences. It is shown in [99] that three highly overrepresented network motifs are present in the transcriptional interaction network of* E. coli*.

## 8. Conclusions

To understand a living organism as a system we first need to understand a cell as a system. This means we need to comprehensively understand the functions of each gene/protein/metabolite and how they work as an individual or in a group. The advancement in molecular biological experiments is producing huge piles of data related to genome and RNA sequence, protein and metabolite abundance, protein-protein interaction, gene expression, and so on. It is important to handle these huge data efficiently and scientifically to understand the cell as a system and to develop new applications in biotechnology and biomedical fields. This, in turn, necessitates the usage of high speed computers and integrating knowledge from other branches of science, for example, statistics, mathematics, physics, chemistry, and so on. The data we need to handle is of old formats, but the present challenge is that it has grown very big and needs the integration of different data types. This can be done by developing efficient scaling techniques for the current software tools and statistical and mathematical models for data handling. The application of network theory and algorithms can facilitate analyzing and integrating big data.

## References

[1] Bell G, Hey T, Szalay A (2009). Computer science: beyond the data deluge. *Science*.

[2] Callebaut W (2012). Scientific perspectivism: a philosopher of science’s response to the challenge of big data biology. *Studies in History and Philosophy of Science C :Studies in History and Philosophy of Biological and Biomedical Sciences*.

[3] Nakamura Y, Afendi FM, Parvin AK (2014). KNApSAcK metabolite activity database for retrieving the relationships between metabolites and biological activities. *Plant and Cell Physiology*.

[4] Kandel ER, Schwartz JH, Jessell TM (2000). *Principles of Neural Science*.

[5] Kanaya S, Yamada Y, Kinouchi M, Kudo Y, Ikemura T (2001). Codon usage and tRNA genes in eukaryotes: correlation of codon usage diversity with translation efficiency and with CG-dinucleotide usage as assessed by multivariate analysis. *Journal of Molecular Evolution*.

[6] Xu Y, Ma P, Shah P, Rokas A, Liu Y, Johnson CH (2013). Non-optimal codon usage is a mechanism to achieve circadian clock conditionality. *Nature*.

[7] Sanger F, Nicklen S, Coulson AR (1977). DNA sequencing with chain-terminating inhibitors. *Proceedings of the National Academy of Sciences of the United States of America*.

[8] Maxam AM, Gilbert W (1977). A new method for sequencing DNA. *Proceedings of the National Academy of Sciences of the United States of America*.

[9] Lander ES, Linton LM, Birren B (2001). Initial sequencing and analysis of the human genome. *Nature*.

[10] Hall N (2007). Advanced sequencing technologies and their wider impact in microbiology. *Journal of Experimental Biology*.

[11] Tucker T, Marra M, Friedman JM (2009). Massively parallel sequencing: the next big thing in genetic medicine. *The American Journal of Human Genetics*.

[12] Ten Bosch JR, Grody WW (2008). Keeping up with the next generation: massively parallel sequencing in clinical diagnostics. *Journal of Molecular Diagnostics*.

[13] Emanuelsson O, Nielsen H, Brunak S, von Heijne G (2000). Predicting subcellular localization of proteins based on their N-terminal amino acid sequence. *Journal of Molecular Biology*.

[14] Zhang Y (2008). Progress and challenges in protein structure prediction. *Current Opinion in Structural Biology*.

[15] Reinharz V, Major F, Waldispühl J (2012). Towards 3D structure prediction of large RNA molecules: an integer programming framework to insert local 3D motifs in RNA secondary structure. *Bioinformatics*.

[16] Wang Z, Xu J (2011). A conditional random fields method for RNA sequence-structure relationship modeling and conformation sampling. *Bioinformatics*.

[17] Laing C, Schlick T (2010). Computational approaches to 3D modeling of RNA. *Journal of Physics Condensed Matter*.

[18] Schena M, Shalon D, Davis RW, Brown PO (1995). Quantitative monitoring of gene expression patterns with a complementary DNA microarray. *Science*.

[19] Lashkari DA, Derisi JL, Mccusker JH (1997). Yeast microarrays for genome wide parallel genetic and gene expression analysis. *Proceedings of the National Academy of Sciences of the United States of America*.

[20] Torres TT, Metta M, Ottenwälder B, Schlötterer C (2008). Gene expression profiling by massively parallel sequencing. *Genome Research*.

[21] Ben-Gal I, Shani A, Gohr A (2005). Identification of transcription factor binding sites with variable-order Bayesian networks. *Bioinformatics*.

[22] Lilley DM (1995). *DNA-Protein: Structural Interactions*.

[23] Ito T, Chiba T, Ozawa R, Yoshida M, Hattori M, Sakaki Y (2001). A comprehensive two-hybrid analysis to explore the yeast protein interactome. *Proceedings of the National Academy of Sciences of the United States of America*.

[24] Rajagopala SV, Sikorski P, Caufield JH, Tovchigrechko A, Uetz P (2012). Studying protein complexes by the yeast two-hybrid system. *Methods*.

[25] Gavin AC, Bösche M, Krause R (2002). Functional organization of the yeast proteome by systematic analysis of protein complexes. *Nature*.

[26] Sparkman OD (2000). Mass spectrometry desk reference. *Journal of the American Society for Mass Spectrometry*.

[27] Palsson BO (2006). *Systems Biology*.

[28] Gentleman R, Carey V, Huber W, Irizarry RA, Dudoit S (2005). *Bioinformatics and Computational Biology Solutions Using R and Bioconductor*.

[29] Kachigan SK (1991). *Multivariate Statistical Analysis: A Conceptual Introduction*.

[30] Rodgers JL, Nicewander WA (1988). Thirteen ways to look at the correlation coefficient. *The American Statistician*.

[31] Verkhedkar KD, Raman K, Chandra NR, Vishveshwara S (2007). Metabolome based reaction graphs of *M. tuberculosis* and *M. leprae*: a comparative network analysis. *PLoS ONE*.

[32] Pereira-Leal JB, Enright AJ, Ouzounis CA (2004). Detection of functional modules from protein interaction networks. *Proteins: Structure, Function and Genetics*.

[33] Kaminuma E, Kosuge T, Kodama Y (2011). DDBJ progress report. *Nucleic Acids Research*.

[34] Benson DA, Karsch-Mizrachi I, Lipman DJ, Ostell J, Wheeler DL (2008). GenBank. *Nucleic Acids Research*.

[35] Asamizu E, Ichihara H, Nakaya A (2014). Plant Genome DataBase Japan (PGDBj): a portal website for the integration of plant genome-related databases. *Plant and Cell Physiology*.

[36] Obayashi T, Okamura Y, Ito S (2014). ATTED-II in 2014: evaluation of gene coexpression in agriculturally important plants. *Plant and Cell Physiology*.

[37] Burge SW, Daub J, Eberhardt R (2013). Rfam 11.0: 10 years of RNA families. *Nucleic Acids Research*.

[38] Kozomara A, Griffiths-Jones S (2014). miRBase: annotating high confidence microRNAs using deep sequencing data. *Nucleic Acids Research*.

[39] The UniProt Consortium (2013). Update on activities at the universal protein resource (UniProt) in 2013. *Nucleic Acids Research*.

[40] Sigrist CJA, de Castro E, Cerutti L (2013). New and continuing developments at PROSITE. *Nucleic Acids Research*.

[41] Bader GD, Betel D, Hogue CWV (2003). BIND: the biomolecular interaction network database. *Nucleic Acids Research*.

[42] Stark C, Breitkreutz B, Reguly T, Boucher L, Breitkreutz A, Tyers M (2006). BioGRID: a general repository for interaction datasets. *Nucleic Acids Research*.

[43] Berman H, Henrick K, Nakamura H, Markley JL (2007). The worldwide protein data bank (wwPDB): ensuring a single, uniform archive of PDB data. *Nucleic Acids Research*.

[44] Kandasamy K, Mohan SS, Raju R (2010). NetPath: a public resource of curated signal transduction pathways. *Genome Biology*.

[45] Shinbo Y, Nakamura Y, Altaf-Ul-Amin M (2006). KNApSAcK: a comprehensive species-metabolite relationship database. *Plant Metabolomics*.

[46] Afendi FM, Okada T, Yamazaki M (2012). KNApSAcK family databases: integrated metabolite-plant species databases for multifaceted plant research. *Plant and Cell Physiology*.

[47] Afendi FM, Ono N, Nakamura Y (2013). Data mining methods for omics and knowledge of crude medicinal plants toward big data biology. *Computational and Structural Biotechnology Journal*.

[48] Hotelling H (1933). Analysis of a complex of statistical variables into principal components. *Journal of Educational Psychology*.

[49] Aldrich J (1998). Doing least squares: perspectives from Gauss and Yule. *International Statistical Review*.

[50] Wilson B (2010). Using PLS to investigate interaction effects between higher order branding constructs. *Handbook of Partial Least Squares*.

[51] Cortes C, Vapnik V (1995). Support-vector networks. *Machine Learning*.

[52] Aleksander I, Morton H (1990). *An Introduction to Neural Computing*.

[53] Mitchell TM (1997). *Machine Learning*.

[54] Breiman L (2001). Random forests. *Machine Learning*.

[55] Afendi FM, Darusman LK, Fukuyama M, Altaf-Ul-Amin M, Kanaya S (2012). A Bootstrapping approach for investigating the consistency of assignment of plants to Jamu efficacy by PLS-DA Model. *Malaysian Journal of Mathematical Sciences*.

[56] Wold S, Sjöström M (1977). SIMCA: a method for analyzing chemical data in terms of similarity and analogy. *Chemometrics: Theory and Application*.

[57] Defays D (1977). An efficient algorithm for a complete link method. *The Computer Journal*.

[58] Sibson R (1973). SLINK: an optimally efficient algorithm for the single-link cluster method. *The Computer Journal*.

[59] MacQueen J Some methods for classification and analysis of multivariate observations.

[60] Kohonen T (1982). Self-organized formation of topologically correct feature maps. *Biological Cybernetics*.

[61] Kanaya S, Kinouchi M, Abe T (2001). Analysis of codon usage diversity of bacterial genes with a self-organizing map (SOM): characterization of horizontally transferred genes with emphasis on the *E. coli* O157 genome. *Gene*.

[62] Takahashi H, Morioka R, Ito R (2011). Dynamics of time-lagged gene-to-metabolite networks of *Escherichia coli* elucidated by integrative omics approach. *OMICS: A Journal of Integrative Biology*.

[63] Morioka R, Kanaya S, Hirai MY, Yano M, Ogasawara N, Saito K (2007). Predicting state transitions in the transcriptome and metabolome using a linear dynamical system model. *BMC Bioinformatics*.

[64] Ikeda S, Abe T, Nakamura Y (2013). Systematization of the protein sequence diversity in enzymes related to secondary metabolic pathways in plants, in the context of big data biology inspired by the KNApSAcK motorcycle database. *Plant and Cell Physiology*.

[65] Junker BH, Schreiber F (2008). *Analysis of Biological Networks*.

[66] Seidman SB (1983). Network structure and minimum degree. *Social Networks*.

[67] Edachery J, Sen A, Brandenburg FJ (1999). Graph clustering using distance-k cliques. *Graph Drawing*.

[68] Matula DW (1972). k-Components, clusters and slicings in graphs. *SIAM Journal on Applied Mathematics*.

[69] Altaf-Ul-Amin M, Shinbo Y, Mihara K, Kurokawa K, Kanaya S (2006). Development and implementation of an algorithm for detection of protein complexes in large interaction networks. *BMC Bioinformatics*.

[70] van Dongen SM (2000). *Graph Clustering by Flow Simulation*.

[71] Girvan M, Newman MEJ (2002). Community structure in social and biological networks. *Proceedings of the National Academy of Sciences of the United States of America*.

[72] Altaf-Ul-Amin M, Wada M, Kanaya S (2012). Partitioning a PPI network into overlapping modules constrained by high-density and periphery tracking. *ISRN Biomathematics*.

[73] Bader GD, Hogue CWV (2003). An automated method for finding molecular complexes in large protein interaction networks. *BMC Bioinformatics*.

[74] Wu M, Li X, Kwoh CK, Ng SK (2009). A core-attachment based method to detect protein complexes in PPI networks. *BMC Bioinformatics*.

[75] Leung HCM, Xiang Q, Yiu SM, Chin FYL (2009). Predicting protein complexes from PPI data: a core-attachment approach. *Journal of Computational Biology*.

[76] Ning K (2009). *Refining Markov Clustering for Protein Complex Prediction by Incorporating Core-Attachment Structure*.

[77] Nishikata K, Wada M, Takahashi H, Nakamura K, Kanaya S, Altaf-Ul-Amin M (2009). Predicting conformation of protein complexes by determining statistically significant domain-domain interactions. *Plant Biotechnology*.

[78] Deng M, Mehta S, Sun F, Chen T (2002). Inferring domain-domain interactions from protein-protein interactions. *Genome Research*.

[79] Kelley BP, Yuan B, Lewitter F, Sharan R, Stockwell BR, Ideker T (2004). PathBLAST: a tool for alignment of protein interaction networks. *Nucleic Acids Research*.

[80] Gunsalus KC, Ge H, Schetter AJ (2005). Predictive models of molecular machines involved in *Caenorhabditis elegans* early embryogenesis. *Nature*.

[81] Matsuda F, Saito K Integrative analysis of secondary metabolism and transcript regulation in *Arabidopsis thaliana*. *The Handbook of Plant Metabolomics*.

[82] Ptacek J, Devgan G, Michaud G (2005). Global analysis of protein phosphorylation in yeast. *Nature*.

[83] Albert R, Jeong H, Barabási AL (2000). Error and attack tolerance of complex networks. *Nature*.

[84] Jeong H, Mason SP, Barabási AL, Oltvai ZN (2001). Lethality and centrality in protein networks. *Nature*.

[85] Junker BH, Koschützki D, Schreiber F (2006). Exploration of biological network centralities with CentiBiN. *BMC Bioinformatics*.

[86] Chen J, Aronow BJ, Jegga AG (2009). Disease candidate gene identification and prioritization using protein interaction networks. *BMC Bioinformatics*.

[87] Nibbe RK, Markowitz S, Myeroff L, Ewing R, Chance MR (2009). Discovery and scoring of protein interaction subnetworks discriminative of late stage human colon cancer. *Molecular & Cellular Proteomics*.

[88] Nibbe RK, Koyutü M, Chance MR (2010). An integrative-omics approach to identify functional sub-networks in human colorectal cancer. *PLoS Computational Biology*.

[89] Eckert T, Tang C, Eidelberg D (2007). Assessment of the progression of Parkinson’s disease: a metabolic network approach. *The Lancet Neurology*.

[90] Lee HS, Bae T, Lee JH (2012). Rational drug repositioning guided by an integrated pharmacological network of protein, disease and drug. *BMC Systems Biology*.

[91] Hu G, Agarwal P (2009). Human disease-drug network based on genomic expression profiles. *PLoS ONE*.

[92] Gottlieb A, Stein GY, Ruppin E, Sharan R (2011). PREDICT: a method for inferring novel drug indications with application to personalized medicine. *Molecular Systems Biology*.

[93] Zhao S, Li S (2010). Network-based relating pharmacological and genomic spaces for drug target identification. *PLoS ONE*.

[94] Huang J, Niu C, Green CD, Yang L, Mei H, Han JDJ (2013). Systematic prediction of pharmacodynamic drug-drug interactions through protein-protein-interaction network. *PLoS Computational Biology*.

[95] Bansal S, Khandelwal S, Meyers LA (2009). Exploring biological network structure with clustered random networks. *BMC Bioinformatics*.

[96] Luscombe NM, Babu MM, Yu H, Snyder M, Teichmann SA, Gerstein M (2004). Genomic analysis of regulatory network dynamics reveals large topological changes. *Nature*.

[97] Kuchaiev O, Stevanović A, Hayes W, Pržulj N (2011). GraphCrunch 2: software tool for network modeling, alignment and clustering. *BMC Bioinformatics*.

[98] Pržulj N, Corneil DG, Jurisica I (2004). Modeling interactome: scale-free or geometric?. *Bioinformatics*.

[99] Milo R, Shen-Orr S, Itzkovitz S, Kashtan N, Chklovskii D, Alon U (2002). Network motifs: simple building blocks of complex networks. *Science*.

